# Homozygous *ALOXE3* Nonsense Variant Identified in a Patient with Non-Bullous Congenital Ichthyosiform Erythroderma Complicated by Superimposed Bullous Majocchi’s Granuloma: The Consequences of Skin Barrier Dysfunction

**DOI:** 10.3390/ijms160921791

**Published:** 2015-09-09

**Authors:** Tao Wang, Chenchen Xu, Xiping Zhou, Chunjia Li, Hongbing Zhang, Bill Q. Lian, Jonathan J. Lee, Jun Shen, Yuehua Liu, Christine Guo Lian

**Affiliations:** 1 Department of Dermatology, Peking Union Medical College Hospital, Peking Union Medical College & Chinese Academy of Medical Sciences, Beijing 100730, China; E-Mails: tombearwt@126.com (T.W.); xuchenchen86@163.com (C.X.); xiping_zhou@126.com (X.Z.); 2 State Key Laboratory of Medical Molecular Biology, Department of Physiology, Institute of Basic Medical Sciences and School of Basic Medicine, Chinese Academy of Medical Sciences and Peking Union Medical College, Beijing 100730, China; E-Mails: jiajialiapple66@163.com (C.L.); hbzhang@ibms.pumc.edu.cn (H.Z.); 3 Department of Medicine, University of Massachusetts, Worcester, MA 01655, USA; E-Mail: Bill.lian@umassmemorial.org; 4 Department of Pathology, Brigham & Women’s Hospital, Harvard Medical School, 221 Longwood Ave. EBRC 401, Boston, MA 02115, USA; E-Mails: jonathan_lee@hms.harvard.edu (J.J.L.); Jshen5@partners.org (J.S.)

**Keywords:** autosomal recessive congenital ichthyosis (ARCI), non-bullous congenital ichthyosiformerythroderma (NBCIE), Whole Exon Sequencing (WES), arachidonatelipoxygenase 3 (ALOXE3), peroxisome proliferator-activated receptor α (PPARα)

## Abstract

Non-bullous congenital ichthyosiform erythroderma (NBCIE) is a hereditary disorder of keratinization caused by pathogenic variants in genes encoding enzymes important to lipid processing and terminal keratinocyte differentiation. Impaired function of these enzymes can cause pathologic epidermal scaling, significantly reduced skin barrier function. In this study, we have performed a focused, genetic analysis of a probrand affected by NBCIE and extended this to his consanguineous parents. Targeted capture and next-generation sequencing was performed on NBCIE associated genes in the proband and his unaffected consanguineous parents. We identified a homozygous nonsense variant c.814C>T (p.Arg272*) in *ALOXE3* (NM_001165960.1) in the proband and discovered that his parents are both heterozygous carriers of the variant. The clinical manifestations of the proband’s skin were consistent with NBCIE, and detailed histopathological assessment revealed epidermal bulla formation and Majocchi’s granuloma. Infection with *Trichophyton rubrum* was confirmed by culture. The patient responded to oral terbinafine antifungal treatment. Decreased skin barrier function, such as that caused by hereditary disorders of keratinization, can increase the risk of severe cutaneous fungal infections and the formation of Majocchi’s granuloma and associated alopecia. Patients with NBCIE should be alerted to the possible predisposition for developing dermatophytoses and warrant close clinical follow-up.

## 1. Introduction

Autosomal recessive congenital ichthyosis (ARCI) represents a group of hereditary disorders of keratinization with clinical manifestations of diffuse, widespread thickened epidermal scaling and varies degree of erythroderma [1]. ARCI is composed of a group of genetically heterogeneous, autosomal recessive genodermatoses that includes three major clinical subtypes, including harlequin ichthyosis (HI; OMIM 242500) as well lamellar ichthyosis (LI; OMIM 242300) and non-bullous congenital ichthyosiform erythroderma (NBCIE; OMIM 242100), the latter two of which often display phenotypic overlap [2,3]. Harlequin ichthyosis represents the most extreme and distinct form of ARCI, named after the dramatic facial features and diamond-shaped scaling of affected neonates, who often die days to weeks after birth due to respiratory insufficiency or sepsis [4]. On the other hand, LI is characterized by large, dark, plate-like epidermal scales with minimal erythema that typically replace the collodion membrane with which an affected neonate is born within. NBCIE can present similarly at birth but, in contrast to LI, is typified clinically by erythroderma and fine, white, overlying scale. Moreover, the clinical features of NBCIE are typically on the milder end of the spectrum [4]. However, patients not infrequently display an overlap of these prototypical dermatologic findings and often fall somewhere within this phenotypic spectrum, making it difficult to classify them as having either NBCIE or LI on strictly clinical grounds. Thus, the current classification system recognizes that LI and NBCIE (or CIE for congenital ichthyosiform erythroderma) are part of a continuum and groups this spectrum of disorders alongside harlequin ichthyosis under within the ARCI group of disorders [3]. All forms of ARCI are associated with significantly impaired skin barrier function, which is primarily due to the inability of terminally differentiated keratinocytes within the epidermis to produce and/or secrete the lipids required for formation of the protective cornified cell envelope and stratum corneum [5]. ARCI, particularly HI, is a potentially life-threatening condition with an estimated prevalence of one in 300,000 newborns [6].

Pathogenic variants in genes encoding enzymes involved in keratinocyte lipid processing, including *ABCA12*, *ALOX12B*, *ALOXE3*, *CERS3*, *CYP4F22*, *NIPAL4*, *PNPLA1*, and *TGM1* [7], have been detected in patients with ARCI. Ultrastructural investigations of keratinocytes in patients with different subtypes of ARCI demonstrate distinct cytomorphologic features that reflect abnormal intracellular lipid processing. Ultimately, this causes alterations in the geometry of lipid packing within the stratum corneum, leading to impaired barrier function, elevated transepidermal fluid loss, and the overall phenotype of this condition. Herein, we describe the clinical, pathologic, and genetic features of the first individual of Chinese extraction diagnosed with NBCIE, which we discovered to be attributable to a homozygous nonsense variant in the *ALOXE3* gene.

## 2. Results

### 2.1. Clinical Presentation and Family History

An 11-year-old boy of Chinese extraction was brought to our clinic by his consanguineous parents (first cousins) for the evaluation of multiple dermatologic issues. The patient lives in an isolated, rural, mountainous region of southern China with limited access to medical care. The patient’s parents recall that the patient’s total body surface appeared “pink and smooth” at birth. Further details regarding the first five years of life were difficult to obtain, given that the child’s parents, his father a coal miner, had little access to the child during this time. However, over the past six years, the boy suffered from severe, diffuse epidermal scaling and, over the past year, had also developed multifocal hair loss and erythema. Initially, the patient had developed red, scaly lesions on the bilateral ankles that eventually progressed to involve the entire body. The patient also suffered from severe xerosis and pruritus, particularly during the winter season that was particularly worse on the ankles, popliteal fossa, and groin. In addition, the patient was also noted to produce limited sweat, even during warmer seasons, and would occasionally be noted to be febrile, despite lacking any sign of underlying infection. The patient had not received any form of systemic or dermatologic treatment prior to his presentation.

**Figure 1:**
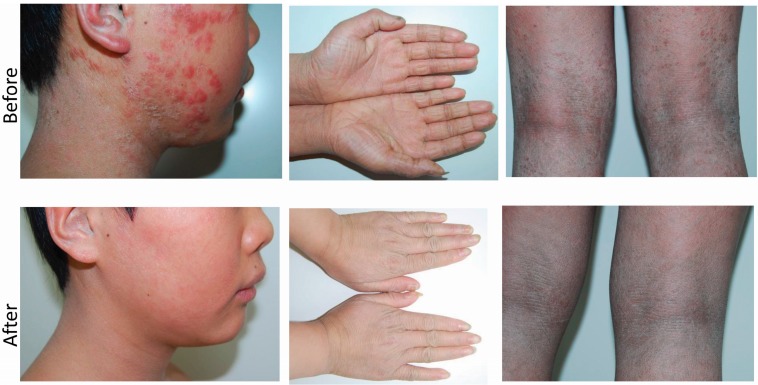
Clinical photos. Erythematous macules and papules involved the face and marked thick scaling of the bilateral palms and popliteal fossae before treatment (**upper row**); Dramatic resolution of the erythema as well as papules and pustules was observed after oral terbinafine treatment (**lower row**).

Upon physical examination, there were multiple, widespread round, erythematous, polycyclic macules with the overlying scale involving 95% of the total body surface area. There was marked, thick, cornification diffusely involving the bilateral palmoplantar surfaces. Erythematous macules and papules with overlying scale were also present on the left scalp, right cheek, and neck, and were noted to have been present for one year (Figure 1). In addition, there were multiple, 2–3 cm areas of hair loss on the left scalp. There was no ectropion or eclabium. Onycholysis, composed of a yellow discoloration, crumbling, and thickening of the first, fourth and fifth fingernails were present. There was no evidence of hearing impairment. His height and weight were within the normal age-appropriate range.

Based on the constellation of clinical and physical exam findings, a preliminary diagnosis of congenital ichthyosis was made. In addition, based on the presence of significant erythema and scaling, there was also concern for an overlying, superficial fungal infection. Thus, a potassium hydroxide preparation, punch biopsies (one from the buttocks and one from the scalp), and tissue cultures were obtained. Moreover, the patient and his parents were consented to enroll in a research protocol approved by the Beijing Genomics Institute at Shenzhen (BGI) to identify potentially clinically-relevant DNA sequence variants in specific targeted genes [8].

### 2.2. Histopathological Examination Confirmed the NBCIE Diagnosis

**Figure 2:**
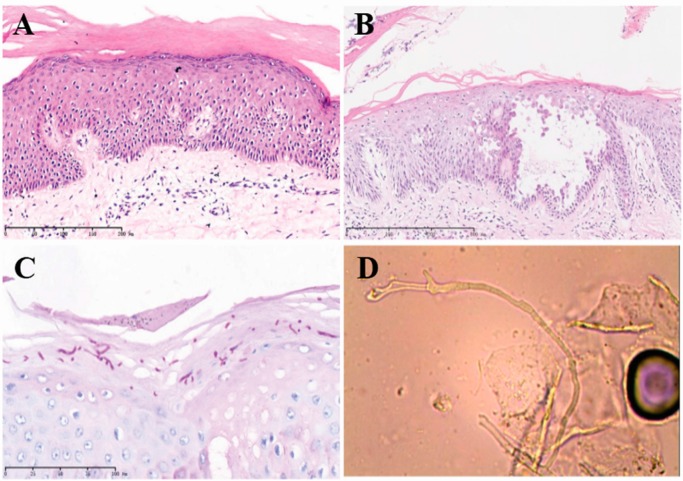
Histopathological findings of biopsy specimens. (**A**,**B**) H and E sections (10X, 40X) of the scalp biopsy showed intraepidermal vesicle formation with acantholytic keratinocytes and overlying scale with parakeratosis; (**C**) Periodic acid-Schiff (PAS) stains highlighted fungal elements; (**D**) *Trichophyton rubrum* was confirmed by culture. Scale bar of **A**, **B** and **C** are in the figures with 200, 800 and 100 µm, respectively.

Microscopic examination of the buttock skin biopsy of H and E stained sections revealed marked hyperkeratosis and focal parakeratosis with mild hypergranulosis within a diffusely acanthotic epidermis (Figure 2A). Acantholytic balloon degeneration and a focus of liquefactive degeneration of the basal cell layer were also present. Direct immunofluorescence was negative for IgG and C3 depositions as were levels of serum antibodies against desmoglein 1 and 3. Taken together, the histologic findings were consistent with non-bullous congenital ichthyosiform erythroderma.

### 2.3. Superimposed Bullous Majocchi’s Granuloma

Microscopic examination of the scalp biopsy showed intraepidermal vesicle formation with acantholytic keratinocytes and overlying scale with parakeratosis (Figure 2B). Periodic acid-Schiff (PAS) stains highlighted fungal elements (Figure 2C). KOH preparation evaluation confirmed the presence of fungal hyphae. DNA extracted from tissue samples of cutaneous scales were amplified by PCR, and subsequent sequencing of the amplification product ultimately detected *Trichophyton rubrum.* The findings were later confirmed by tissue culture (Figure 2D).

### 2.4. A Homozygous Nonsense Variant in ALOXE3 Was Identified by Genomic Analyses

**Figure 3:**
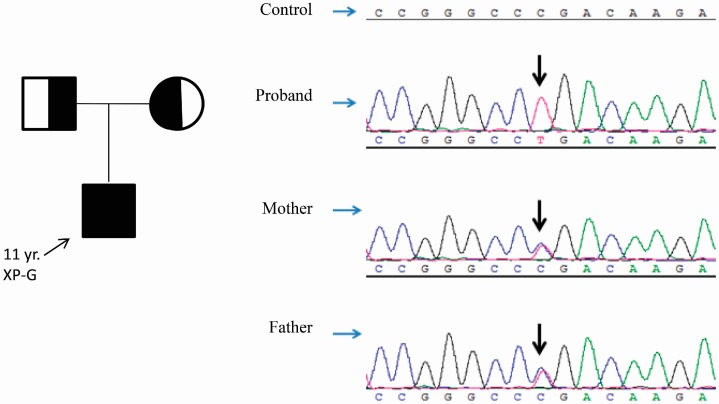
Pedigree chart of the NBCIE family with the *ALOXE3* variant. Sanger sequencing confirmed the c.814C>T (p.(Arg272*)) variant in the *ALOXE3* (NM_001165960.1) gene in the proband and his consanguineous parents. The chromatograms are shown and the arrows point to the variants. The proband is homozygous for the variant and his parents are heterozygous carriers.

Targeted next-generation sequencing and confirmation by Sanger sequencing revealed the c.814C>T (p.Arg272*) variant in *ALOXE3* (NM_001165960.1) within this consanguineous family (Figure 3). The proband was homozygous for the variant, and his parents were both heterozygous carriers (Figure 3). Based on the consanguineous marriage history of the patient’s parents, these results are consistent with autosomal recessive inheritance. The C to T transition at position 814 in exon 4 of *ALOXE3* cDNA changes the “CGA” codon at position 272, normally encoding an arginine residue, to the “TGA” stop codon, which is predicted to lead to a truncated or absent protein. This variant has been reported (dbSNP ID rs370031870) in 1 out of 4406 African American chromosomes by the National Hart, Lung, and Blood Institute Exome Sequencing Project (NHLBI ESP, http://evs.gs.washington.edu/EVS/) and 1 out of 10,404 African chromosomes by the Exome Aggregation Consortium (ExAC, http://exac.broadinstitute.org) (Table 1). This low frequency in the general population is consistent with the carrier frequency for a rare recessive trait.

**Table 1:** Summary of reported variants in *ALOXE3* (including the variant identified in our non-bullous congenital ichthyosiform erythroderma (NBCIE) patient of this study in bold). Number of reported alleles refers to those found in affected individuals. The g. nomenclature is based on GRCh37 (hg19) human reference sequence NC_000017, the c. nomenclature is based on the cDNA sequence NM_001165960.1, in which the “A” in the “ATG” start codon is denoted number 1, and the p. nomenclature is based on the translated protein. The variant documented in the proband is emboldened and italicized.

gDNA	cDNA	Protein	Exon	# of Reported Alleles	Allele Frequency in ExAC
g.8020119G>T	c.723C>A [9]	p.(Cys241 *)	3	1	0
***g.8018941G>A***	***c.814C>T***	p.(Arg272 *)	***4***	***2***	***1/121354 (rs370031870)***
g.8018925C>T	c.830G>A [10]	p.(Arg277fs *12)	4	2	3/121250
g.8015495G>A	c.1096C>T [9,10,11,12,13]	p.(Arg366 *)	7	15	14/121390 (rs121434233)
g.8015476delT	c.1115delA [10]	p.(Lys372fs*40)	7	1	0
g.8014792C>A	c.1238G>T [11]	p.(Gly413Val)	7	1	0
g.8013765_8013773del	c.1427_1435del9 [10]	p.(Gln476_Ala479delinsPro)	9	2	0
g.8013529G>T	c.1582C>A [12]	p.(Arg528Ser)	10	2	0
g.8013435A>G	c.1676T>C [9]	p.(Leu559Pro)	10	2	0
g.8012556C>A	c.1894G>T [12]	p.(Val632Phe)	11	6	0
g.8011840G>A	c.2026C>T [11]	p.(Gln676*)	13	2	6/121282
g.8006708G>A	c.2285C>T [9,10,11,12]	p.(Pro762Leu)	15	18	114/121404 (rs147149459)
g.8013409C>A	c.1701+1G>T [13]		Intron 10	1	0
g.8013408A>T	c.1701+2A>T [13]		Intron 10	1	0

### 2.5. Treatment and Response

The patient received a two-week course of oral terbinafine, which led to dramatic resolution of the erythema as well as papules and pustules (Figure 1 and Figure 2). A topical glycerol based emollient was prescribed for other affected regions. At follow-up visit ten weeks after his initial presentation, the patient exhibited significant improvement in the regions affected by dermatophytic infection as well as in the scaly, xerotic regions involved by underlying ARCI. Areas of hair loss had also significantly improved.

## 3. Discussion

We describe the case of a pediatric patient of Chinese extraction, born to consanguineous parents, who was diagnosed with autosomal recessive congenital ichthyosis of the NBCIE subtype. Our sequencing results revealed that the genodermatosis was caused by a homozygous nonsense variant c.814C>T (p.(Arg272*)) in *ALOXE3*, which was complicated by a superimposed *Trichophyton rubrum* infection and associated alopecia. *ALOXE3* encodes the arachidonate lipoxygenase 3 enzyme, which functions as a hydroperoxide isomerase that oxidizes the 9R-hydroperoxide derivative of esterified ω-hydroxyacyl-sphingosine (EOS) to synthesize a unique epoxy alcohol important for activating the nuclear receptor peroxisome proliferator-activated receptor α (PPARα). This pathway is critical for proper epidermal differentiation. The *ALOXE3* gene is located on chromosome 17p13.1 and contains 15 exons [14]. That it is predominantly expressed in the suprabasal layers of the epidermis supports its role in the advanced phases of epidermal differentiation and in lamellar body processing [1]. These enzymes act in tandem to oxidize the linoleic acid group, which is esterified to ω-hydroxyacyl ceramide and produced within the keratinocyte [15,16,17,18]. This important set of enzymatic reactions facilitates the ultimate hydrolysis of the oxidized linoleate from ceramide, which is then released and free to bind proteins of the cornified envelope, thereby helping form a key component of the epidermal barrier [16]. We speculate that the homozygous, nonsense germline variant harbored by this patient resulted in loss of this key enzymatic mechanism and, therefore, the loss of proper keratinocyte differentiation, lipid metabolism, and epidermal barrier function. This underlying substrate, in addition to limited access to proper hygiene and medical care, compounded by the warm, humid environment, likely predisposed the patient to develop this severe, superimposed dermatophytic infection.

Thirteen variants in *ALOXE3* have been reported since the gene was first identified as an ARCI gene in 2002 [10,11,12]. These known variants contribute to approximately 15% of ARCI cases (Table 1) [10,19]. Jobard *et al.* described two point mutations and one deletion found in *ALOXE3* in a family of consanguineous lineage from the Mediterranean basin [12]. Eckl *et al.* described the molecular and clinical findings in 17 families with ARCI originating from Central Europe, Turkey, and the Indian subcontinent, and identified four different inactivating variants in *ALOXE3* [11]. In addition, Eckl*et et al.* also found 11 previously unreported variants in *ALOX12B* and *ALOXE3* in 21 ARCI patients from 19 unrelated families and demonstrated that variants in these two genes were the second most common cause for ARCI in their cohort of patients [10]. Examination of the molecular data revealed allelic heterogeneity for *ALOX12B* and two mutational hotspots in *ALOXE3* in 2009 [10]. Vahlquist *et al.* also identified three patients with germline variants in *ALOXE3*: one Swedish patient was homozygous for a T>C transition at cDNA position 1676 in exon 10 (p.(Leu559Pro)) while the others were compound heterozygotes [9]. Patients with pathogenic variants in the *ALOXE3* and *ALOX12B* genes usually present with a NBCIE phenotype [11,12,20]. The scaling is typically mild to moderate in severity and takes on a whitish or light brown hue. Up to 76% of individuals with ARCI are born as collodion babies and 88% have sweating disorders [10]. Patients with pathogenic variants in the *ALOX12B* gene show more limited, whitish desquamation compared with those with *ALOXE3* variants. In our case, the patient exhibited scaling of a grey to dark hue.

Taken together, our patient’s phenotype, including the presence of generalized erythema and the thick dark scaling over the joints in our patient, was reflective of his germline homozygous nonsense c.814C>T (p.(Arg272*)) variant in the *ALOXE3* gene. The variant found in our patient has not been reported in the Human Gene Mutation Database and represents the results of serendipitous contributions from consanguineous parents, who were both carriers. The patient presented to us with severe ichthyosiform skin lesions and was ultimately diagnosed with NBCIE. However, unlike most existing reported cases of ACRI, we identified multiple microscopic, inner epidermal bullae, which were negative on direct and indirect immunofluorescence studies. We hypothesize that the robust inflammatory reaction, secondary to heavy dermatophyte infestation led to the formation of intraepidermal bullae in our ARCI patient. In addition, the patient’s alopecia resolved after anti-fungal therapy, implying that this manifestation was secondary to the infection and not the underlying ichthyosis. Furthermore, we speculate that the homozygous nonsense variant in *ALOXE3* led to severe impairment in skin barrier function, which predisposed our patient to this severe cutaneous fungal infection.

## 4. Experimental Section

### 4.1. Ethics Statement

The study was conducted in accordance with the guiding principles of the Declaration of Helsinki. Collection of samples was approved by the ethical committees of Peking Union Medical College Hospital, and informed written consent was obtained from the parents. The parents completely understood that their peripheral blood samples would be used in this research and requested genetic counseling. We followed all regulations for the enrollment of patients, sample collection and informed consent for the purpose of research.

### 4.2. Histopathological Examination and Special Stains

Routine histology was performed by microscopic examination of 5 µm Hematoxylin and Eosin (H&E) stained sections of the skin biopsy obtained from the buttock region. Direct immunofluorescence was performed to evaluate for IgG and C3 deposition. Fungal elements were detected by potassium hydroxide (KOH) preparation as well as Periodic acid-Schiff (PAS) special stains.

### 4.3. Serological Studies

Serum antibodies for desmoglein 1 and 3 detection were performed with standard laboratory testing (OuMeng, Beijing, China).

### 4.4. Genomic DNA Extraction

Genomic DNA was extracted from peripheral blood samples of the patient’s family members using QIAamp Blood DNA mini Kit (Qiagen, Hilden, Germany) according to the manufacturer’s instructions. After quality control via gel electrophoresis, 1 µg of high molecular weight genomic DNA sample was randomly fragmented to approximately 250 bp using a Covaris focused-ultrasonicator. Next, Gel-purified DNA was end-repaired with T4 DNA polymerase, T4 phosphonucleotide kinase and the Klenow fragment of *Escherichia coli* DNA polymerase to fill 5′ overhangs and remove 3′ overhangs. Terminal A residues were added following a brief incubation with dATP and the Klenow 3′–5′ exo-enzyme following standard Illumina protocols. Subsequently, adapters were ligated to both ends of the resulting fragments. The adapter-ligated templates were purified by the Agencourt AMPure SPRI beads.

### 4.5. Targeted Next Generation Sequencing (NGS) of NBCIE-Associated Genes

A 2.1M Human Array targeting seven NBCIE disease-causing genes encoding exons and 10 bp flanking intronic sequences was designed with Roche NimbleGen (Madison, WI, USA). The adapter-ligated DNA templates were amplified by ligation-mediated polymerase chain reaction (LM-PCR), purified, pooled, and hybridized to the array for enrichment. Captured ligation mediated-PCR products were subjected to Agilent 2100 Bioanalyzer and real-time quantitative PCR on ABI StepOne Real-time PCR system (Life technologies, Grand Island, NY, USA) to estimate the magnitude of enrichment. Qualified library was sequenced by Illumina HiSeq2000 Analyzers (following the manufacturer’s protocols) for 90 cycles per read to generate paired-end reads. Image analysis, error estimation, and base calling were performed using Illumina Pipeline software (version 1.3.4) to generate raw data. The “clean reads” derived from targeted sequencing and filtering were then aligned to the human reference genome (hg19/GRCh37) using the BWA (Burrows Wheeler Aligner) Multi-Vision software package. We used SOAPsnp software and Samtools to detect single nucleotide variants and indels [21].

### 4.6. Verification of Variants

Sanger sequencing was used to determine whether any of the variants co-segregated with the disease phenotype in the patient’s family. Primers were designed using Primer 6.0 (www.premierbiosoft.com) and synthesized by BGI-Beijing, Beijing, China. All potential variants identified by NGS of the affected individual were confirmed by Sanger sequencing. Sequencing reactions were performed by mixing 5 μL of purified PCR products (ExoSAP-IT, USB, Cleveland, OH, USA), 0.75 µM of primers and 1 μL of BigDye Terminator v1.1 Cycle Sequencing kit (Applied Biosystems, Foster City, CA, USA) and were run in an ABI 3730 Genetic Analyzer (Applied Biosystems).

## 5. Conclusions

In summary, we present a case of genetically-confirmed NBCIE complicated by a severe, bullous dermatophyte infection and associated alopecia, which was resolved after anti-fungal therapy. Our analysis revealed that the patient harbored a homozygous nonsense variantc.814C>T (p.(Arg272*)) in *ALOXE3*, which we believe ultimately predisposed him to this severe cutaneous infection. Our case also illustrates the importance of the *ALOXE3* gene function throughout evolution while demonstrating the clinical practice of precision medicine through the use of next-generation sequencing technologies.
